# Molecular Regulation of Biosynthesis of Long Chain Polyunsaturated Fatty Acids in Atlantic Salmon

**DOI:** 10.1007/s10126-022-10144-w

**Published:** 2022-07-30

**Authors:** Alex K. Datsomor, Gareth Gillard, Yang Jin, Rolf E. Olsen, Simen R. Sandve

**Affiliations:** 1 Center for Integrative Genetics (CIGENE), Department of Animal and Aquacultural Sciences, Faculty of Biosciences, Norwegian University of Life Sciences, Ås, Norway; 2 Institute of Biology, Norwegian University of Science and Technology, 7491 Trondheim, Norway

**Keywords:** Polyunsaturated fatty acids, Enzymology, Nutritional regulation, Transcriptional control, Genome duplication, Atlantic salmon

## Abstract

Salmon is a rich source of health-promoting omega-3 long chain polyunsaturated fatty acids (n-3 LC-PUFA), such as eicosapentaenoic acid (EPA, 20:5n-3) and docosahexaenoic acid (DHA, 22:6n-3). The LC-PUFA biosynthetic pathway in Atlantic salmon is one of the most studied compared to other teleosts. This has largely been due to the massive replacement of LC-PUFA-rich ingredients in aquafeeds with terrestrial plant oils devoid of these essential fatty acids (EFA) which ultimately pushed dietary content towards the minimal requirement of EFA. The practice would also reduce tissue content of n-3 LC-PUFA compromising the nutritional value of salmon to the human consumer. These necessitated detailed studies of endogenous biosynthetic capability as a contributor to these EFA. This review seeks to provide a comprehensive and concise overview of the current knowledge about the molecular genetics of PUFA biosynthesis in Atlantic salmon, highlighting the enzymology and nutritional regulation as well as transcriptional control networks. Furthermore, we discuss the impact of genome duplication on the complexity of salmon LC-PUFA pathway and highlight probable implications on endogenous biosynthetic capabilities. Finally, we have also compiled and made available a large RNAseq dataset from 316 salmon liver samples together with an R-script visualization resource to aid in explorative and hypothesis-driven research into salmon lipid metabolism.

## General Introduction

**Fig. 1:**
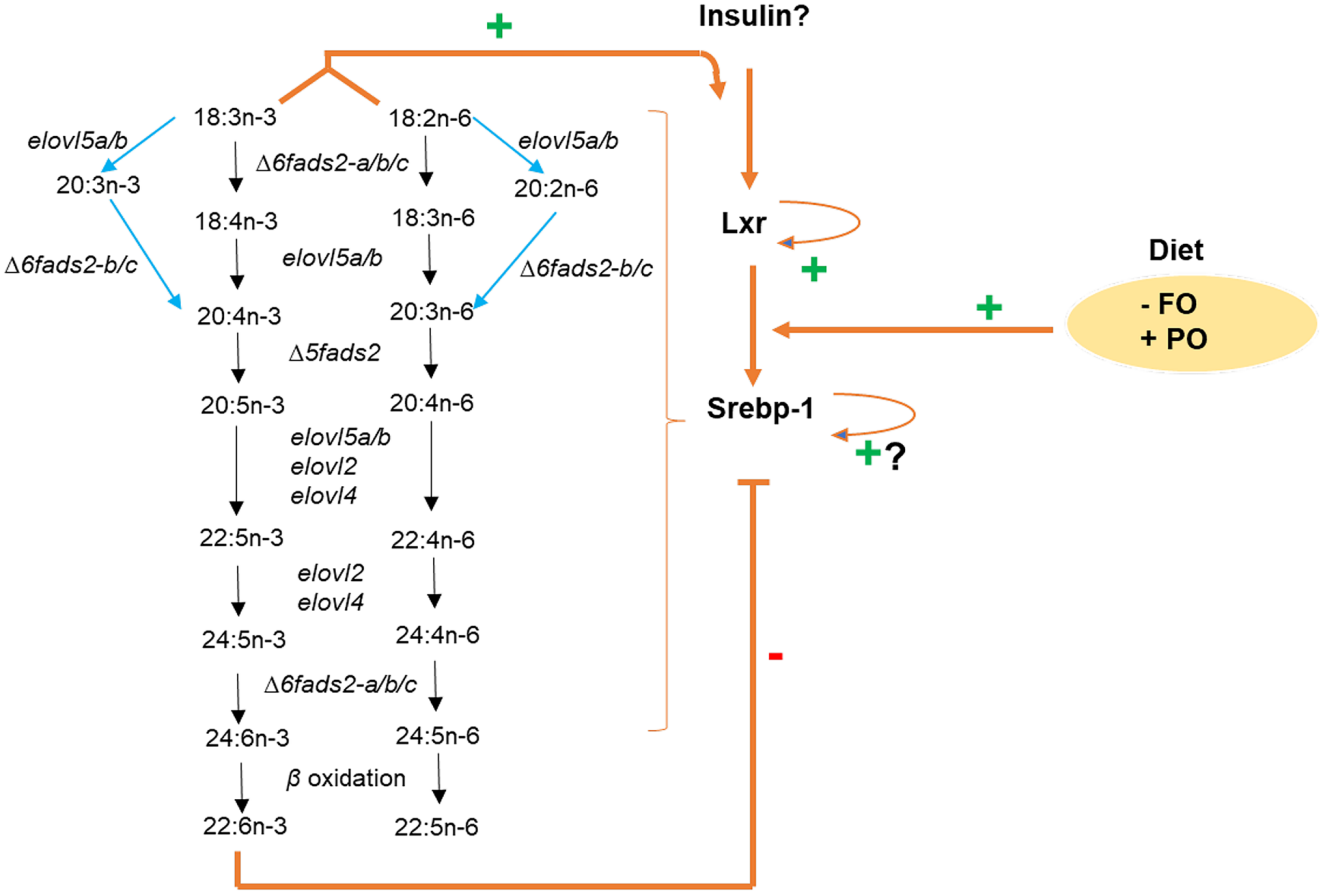
Transcriptional control of Atlantic salmon LC-PUFA biosynthetic pathway: interplay between PUFAs and the Lxr-Srebp-1 transcription regulatory pathway. Activities of enzymes in salmon LC-PUFA biosynthetic pathway have previously been assayed in vitro via heterologous expressional studies in *Saccharomyces cerevisiae* and to some extent in vivo for *∆6fads2-a* (Datsomor et al. 2019a; Zheng et al. 2005), *∆6fads2-b* and *∆6fads2-c* (Monroig et al. 2010), and *∆5fads2* (Hastings et al. 2004), and for the elongases *elovl5 *(*elovl5a* and *elovl5b*) (Hastings et al. 2004; Morais et al. 2009) and *elovl2* (Datsomor et al. 2019b; Morais et al. 2009). Heterologous expression studies have demonstrated that salmon ∆6 Fads2-b and ∆6 Fads2-c possess ∆8-desaturation activities (Monroig et al. 2011a, b), and similar results were obtained from in vivo CRISPR/Cas9 functional studies (Datsomor et al. 2019a). The ∆8-pathway is marked with blue arrows. Results so far suggest possible existence of the liver-x receptor (Lxr)-sterol regulatory element binding protein-1 (Srebp-1) pathway that controls salmon LC-PUFA biosynthetic pathway (Carmona-Antoñanzas et al. 2014; Minghetti et al. 2011). High dietary composition of the PUFA precursors, 18:3n-3 and 18:2n-6 (typical of plant oil–based diets), has been shown to induce the LC-PUFA pathway by increasing desaturation/elongation of PUFAs (Tocher et al. 2003) (denoted as a green plus sign), while high dietary levels of 20:5n-3 and especially 22:6n-3 (typical of fish oil–based diets) inhibit the LC-PUFA biosynthetic pathway (Minghetti et al. 2011) possibly via the Lxr-Srebp-1-dependent fashion (Carmona-Antoñanzas et al. 2014; Datsomor et al. 2019b; Jin et al. 2020a, b; Minghetti et al. 2011); this is depicted as a red minus sign. Treatment of Atlantic salmon SHK-1 cells with GW3965, a potent and selective Lxr agonist induced *lxrα* expression suggesting possible autoregulation (Carmona-Antoñanzas et al. 2014). Furthermore, an analysis of putative transcription factor binding sites within salmon *srebp-1* promoter revealed the presence of sterol regulatory element binding protein site (Grønvold et al. 2021; Samy et al. 2017), also suggesting probable self-regulation

Long-chain polyunsaturated fatty acids (LC-PUFA, ≥ C_20_) are critical for maintaining normal cellular functions in all organisms, yet only few organisms can synthesize these molecules de novo. In vertebrate species, including humans, acquisition of LC-PUFA through feed is therefore essential, which can be supplemented by endogenous biosynthesis from shorter PUFA precursor molecules, albeit at limited rate (Burdge 2019). The biosynthetic capability varies both between and within species and depends largely on the presence of genes encoding enzymes involved in the synthesis of such fatty acids (Monroig et al. 2013). Currently, the increasing growth of human population together with overexploitation of the traditional capture fisheries makes farmed fish particularly salmon a key source of n-3 LC-PUFA (Béné 2015). Additionally, salmon farming also has significant socio-economic impact in many countries and coastal communities providing 132,600 jobs globally with a first-hand value creation of $15.4 billion (USD) per annum (ISFA 2018). However, while farmed salmon is a good source of n-3 LC-PUFA to human consumers, the recent inclusion of terrestrial plant oils devoid of n-3 LC-PUFA in aquafeeds has been shown to ultimately compromise the nutritional value of salmon to the human consumer. Atlantic salmon shows capabilities of synthesizing LC-PUFA from precursors in plant oils (although to a limited degree) as it possesses fatty acyl desaturases (*Fads*) that catalyze introduction of double bonds in fatty acyl chains and elongases of very long chain fatty acids (Elovl) which catalyze carbon chain extension of fatty acids (Fig. 1). In efforts to optimize synthesis of LC-PUFAs from precursors in plant oils, the biosynthetic pathway in salmon has been extensively studied. The genome of the ancestor of Atlantic salmon (*Salmo salar* L.) has undergone a relatively recent whole genome duplication referred to as the salmonid-specific whole genome duplication (Ss4R WGD) ~ 80–100 million years ago (mya) (Lien et al. 2016; Macqueen and Johnston 2014). This has resulted in a large repertoire of LC-PUFA biosynthetic genes that demonstrate varying degrees of functional redundancy (Monroig et al. 2010; Morais et al. 2009) and even bifunctionality (Oboh et al. 2017; Zheng et al. 2005; Monroig et al. 2011a, b). Although molecular mechanisms underlying LC-PUFA biosynthesis in teleosts, including salmonids, have been periodically reviewed (Miller et al. 2008; Monroig et al. 2011a, b; Tocher 2015; Turchini et al. 2009; Xie et al. 2021; Monroig et al. 2011b), an in-depth review of molecular genetics of PUFA biosynthesis (enzyme activity, substrate preferences, and gene repertoire), the regulation of this pathway across life stages, and upon changes in dietary composition is lacking. Here, we review these aspects of the salmon LC-PUFA biosynthesis, including recent advances from large-scale omics data and CRISPR-based approaches, and identify key knowledge gaps that should be addressed in future studies. In this review, we have also compiled and made available a large RNAseq dataset from 316 salmon liver samples as well as R-script visualization resource to aid in explorative and hypothesis-driven research into salmon LC-PUFA biosynthesis.

## Salmon LC-PUFA Biosynthetic Pathways

### LC-PUFA Biosynthetic Enzymes

Like mammals and other teleosts, salmon cannot synthesize PUFA de novo due to the lack of ∆12 and ∆15 fatty acyl desaturases. Instead, LC-PUFA biosynthesis proceeds via consecutive alternating desaturation and elongation of the 18-carbon (C_18_) dietary precursors, linoleic acid (18:2n-6), and α-linolenic acid (18:3n-3) (Fig. 1). While mammals have FADS1 and FADS2 which catalyze ∆5- and ∆6- desaturation, respectively, salmon possess only Fads2 which catalyzes both ∆5- and ∆6- desaturation (Monroig et al. 2010). Four salmon *fads2* genes (*∆6fads2-a*, *∆6fads2-b*, *∆6fads2-c*, and *∆5fads2*) (Hastings et al. 2004; Monroig et al. 2010; Zheng et al. 2005) and four *elovl* genes (*elovl2*, *elovl4*, *elovl5a*, and *elovl5b*) (Carmona-Antoñanzas et al. 2011; Hastings et al. 2004; Morais et al. 2009) have been isolated and functionally characterized by in vitro heterologous expressional studies (Carmona-Antoñanzas et al. 2011; Hastings et al. 2004; Monroig et al. 2010; Morais et al. 2009; Zheng et al. 2005). In these studies, *Saccharomyces cerevisiae* was transformed with expression vectors containing the open reading frame of each gene and then incubated with various potential PUFA substrates, and the proportion of substrates converted to LC-PUFAs was determined using gas chromatography. Additionally, *∆6fads2-a*, *∆6fads2-b*, *∆6fads2-c*, *∆5fads2*, and *elovl2* have further been studied via in vivo CRISPR/Cas9-mediated partial knockouts (Datsomor et al. 2019a; Datsomor et al. 2019b). Salmon Elovl5b amino acid sequence is 91% identical to Elovl5a but 70% identical to many mammalian (rat, mouse, and human) ELOVL5-like proteins (Morais et al. 2009). The salmon Elovl2 on the other hand has amino acid sequence that is 79% identical to zebrafish Elovl2-like protein and 68–71% identical to rat, mouse, and human ELOVL2-like proteins (Morais et al. 2009). The amino acid sequences of salmon ∆6 Fads2-b and ∆6 Fads2-c are 96.7% identical to each other but are 90.4–92.8% identical to ∆6 Fads2-a and ∆5 Fads2 (Monroig et al. 2010). Furthermore, the amino acid sequences of salmon ∆6 Fads2-b and ∆6 Fads2-c have 63.7–63.9% and 56.6–57.3% identity to human ∆6 FADS2 and ∆5 FADS1, respectively (Monroig et al. 2010). The desaturases encoded by all salmon *∆6fads2* predominantly possess ∆6-desaturation activities towards 18:2n-6 and 18.3n-3 (Datsomor et al. 2019a; Monroig et al. 2010) in the rank order of ∆6 Fads2-a > ∆6 Fads2-b > ∆6 Fads2-c (Monroig et al. 2010). Furthermore, ∆6 Fads2-a demonstrates high capacity for ∆6-desaturation of C_24_ substrates, such as 24:4n-6 and 24:5n-3 (Oboh et al. 2017), and also possesses ∆5-desaturation capabilities towards 20:4n-3, but to a low extent (Zheng et al. 2005). The ∆5 Fads2 in salmon predominantly catalyzes ∆5-desaturation of 20:4n-3 with minimal activity towards 20:3n-6 (Hastings et al. 2004). Similar to ∆6 Fads2-a, ∆5 Fads2 demonstrates clear ∆6-desaturation activities towards 24:4n-6 and 24:5n-3 (Oboh et al. 2017) but limited capabilities towards C_18_ ∆6-substrates, such as 18:2n-6 and 18:3n-3 (Hastings et al. 2004). Taken together, Atlantic salmon ∆6 Fads2-a and ∆5 Fads2 demonstrate ∆6/∆5 bifunctional enzyme activities similar to that of zebrafish (*Danio rerio*) ∆6/∆5 Fads2 (Hastings et al. 2001). The two Elovl5 elongases encoded by salmon *elovl5a* and *elovl5b* originate from the salmonid-specific fourth whole genome duplication (Ss4R) which occurred before speciation of salmonids (Carmona-Antoñanzas et al. 2016). Both elongases efficiently elongate the C_18_ substrates, 18:4n-3 and 18:3n-6, and the C_20_ substrates, 20:5n-3 and 20:4n-6, with limited activities towards C_22_ LC-PUFAs (Hastings et al. 2004; Morais et al. 2009). It is also likely that salmon Elovl5s are responsible for elongation of 18:3n-3 and 18:2n-6 to products that proceed through the ∆8-pathway (Fig. 1) which appears to be active in Atlantic salmon (Datsomor et al. 2019a; Monroig et al. 2011a, b). The Elovl2 in salmon shows limited capabilities for 18:4n-3 and 18:3n-6 elongation but higher activities towards 20:5n-3, 20:4n-6, 22:5n-3, and 22:4n-6 (Datsomor et al. 2019b; Morais et al. 2009). Similar to Elovl2, the elongase encoded by salmon *elovl4* efficiently elongates C_20_ and C_22_ LC-PUFAs (Carmona-Antoñanzas et al. 2011) and possesses capacity for converting these fatty acids further to the so-called very long-chain fatty acids (up to C_36_) (Carmona-Antoñanzas et al. 2011). Except for Elovl5b, accumulating evidence from both in vitro and in vivo functional studies shows that enzymes of Atlantic salmon LC-PUFA biosynthetic pathway have preferences for n-3 PUFA compared to their n-6 counterparts (Carmona-Antoñanzas et al. 2011; Datsomor et al. 2019a; Datsomor et al. 2019b; Hastings et al. 2004; Monroig et al. 2011a, b; Monroig et al. 2010; Morais et al. 2009; Oboh et al. 2017; Zheng et al. 2005) and the Elovls show significant functional redundancies similar to the desaturases even though such overlapping functions may vary between tissues depending on expression levels of respective genes. The contribution of the salmonid-specific fourth WGD and perhaps lineage-specific gene duplications to such observed number of enzyme copies and levels of overlapping functions is discussed in the “Impact of Gene Duplication on Evolution of Atlantic Salmon LC-PUFA Biosynthetic Pathway” section.

### Alternative LC-PUFA Biosynthetic Pathways

The classical pathway for LC-PUFA synthesis in salmon has been thought to primarily involve alternating ∆6/∆5 desaturation and elongation steps. However, there is also evidence to support the presence of an unconventional but active ∆8-pathway for bioconversion of 18:3n-3 and 18:2n-6 precursors to LC-PUFA, where synthesis of 20:4n-3 and 20:3n-6 proceeds through elongation of the C_18_ substrates to 20:3n-3 and 20:2n-6 followed by ∆8-desaturation (C_18_ elongation ► C_20_ ∆8-desaturation ► 20:4n-3 and 20:3n-6) similar to what is found in other teleosts (Fig. 1) (Datsomor et al. 2019a; Monroig et al. 2011a, b). In in vitro assays, salmon ∆6 Fads2-b demonstrates clear ∆8-desaturation of the ∆8 substrates, 20:3n-3 and 20:2n-6, while ∆6 Fads2-c shows only limited ∆8-desaturation capacity for 20:3n-3 (Monroig et al. 2011a, b). These results were supported by indirect evidence from CRISPR/Cas9-mediated editing (partial knockout) of salmon ∆6 Fads2-b and ∆6 Fads2-c, which resulted in accumulation of the ∆8 substrates, 20:3n-3 and 20:2n-6, in the liver and white muscle when fish were fed a diet rich in the C_18_ precursors, 18:3n-3 and 18:2n-6 (Datsomor et al. 2019a). Taken together, it is possible that the efficient endogenous biosynthesis of LC-PUFAs in salmon depends not only on the presence of the complementary desaturase and elongase enzymes but also on the occurrence of overlapping functions and bifunctionalities evolved by these enzymes. Though several forms of evidence suggest ∆8-desaturation activities by ∆6 Fads2 enzymes in salmon (Fig. 1) (Datsomor et al. 2019a; Monroig et al. 2011a, b), a better understanding of this pathway will require detailed evaluation of Elovl5-mediated elongation of the C_18_ substrates through in vitro or in vivo assays. It is noteworthy that salmon fed diets rich in 18:3n-3 and 18:2n-6 have high tissue contents of the elongated products (∆8-desaturation substrates), 20:3n-3 and 20:2n-6 (Datsomor et al. 2019a; Morais et al. 2012; Tocher et al. 2003), suggesting a probable Elovl5-mediated elongation of the C_18_ precursors.

## Impact of Gene Duplication on Evolution of Atlantic Salmon LC-PUFA Biosynthetic Pathway

**Fig. 2:**
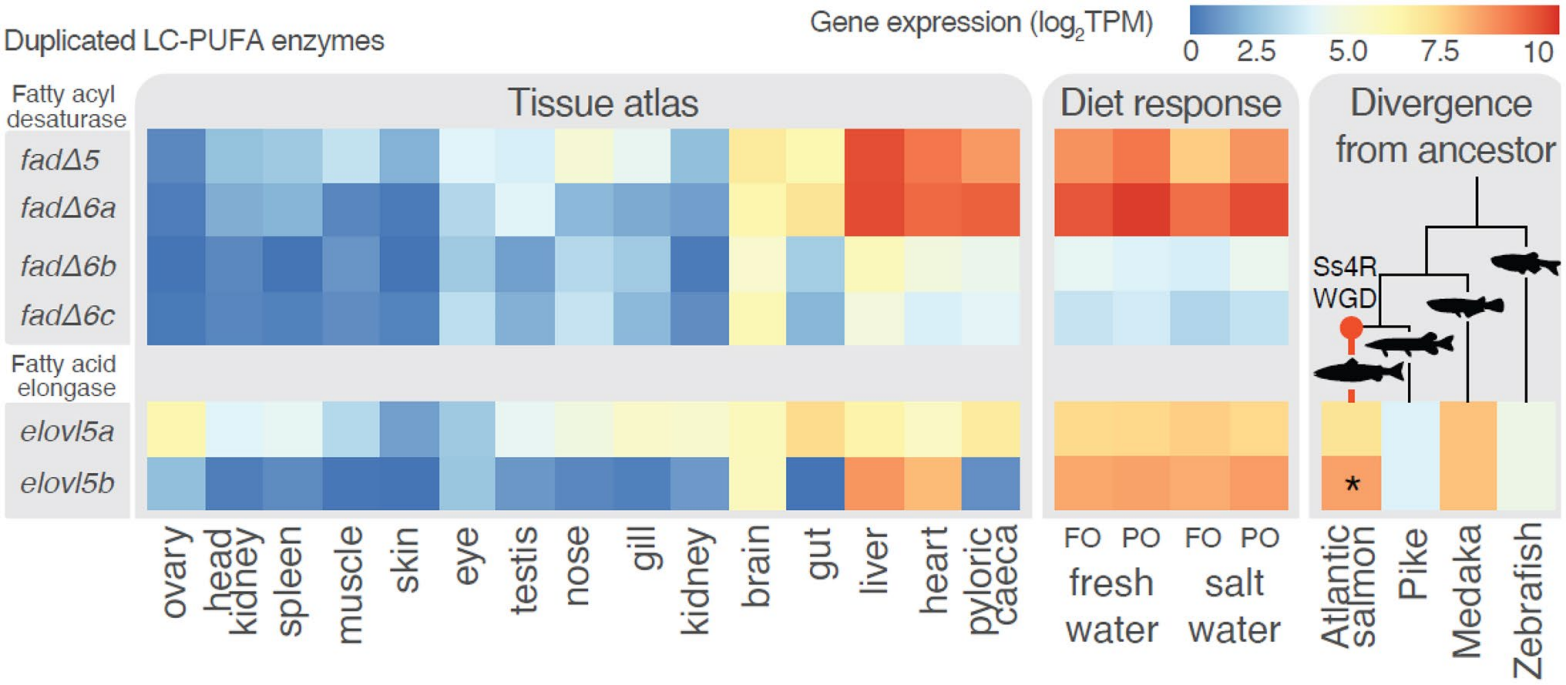
Comparing expression profiles of duplicated LC-PUFA enzymes; delta (Δ) 5 and 6 fatty acyl desaturases (*fads*); and the fatty acid elongases *elovl5a* and *elovl5b* genes. Heatmap shows gene expression levels measured in Transcripts Per Million (TPM) on a log_2_ scale, with the lowest values in blue and the highest in red. A common scale is used across genes and samples. Samples are from three experiments: first, tissue atlas data from Lien et al. 2016 (Lien et al. 2016) shows expression across 15 tissue types, where kidney indicated in the figure excludes the head kidney. Second, data from Gillard et al. 2018 (Gillard et al. 2018) shows liver expression response to two diets, fish oil (FO)– or plant oil (PO)–based diet, at two life stages, in freshwater or saltwater (mean of the day 20 samples). Lastly, data from Gillard et al. 2021 (Gillard et al. 2021) shows liver expression of *elovl* in Atlantic salmon compared to species without the salmonid-specific whole genome duplication (Ss4R WGD). Salmon *elovl5b* was found to have significantly (*p < 0.05) diverged from the ancestral state, with higher expression in salmonid livers

The ancestor of all salmonids experienced a relatively recent WGD ~ 80–100 million years ago (Macqueen and Johnston 2014; Lien et al. 2016). In Atlantic salmon ~ 50% of the resulting gene duplicates are still retained and expressed at the genome wide level, and about 50% of these have evolved some level of novel regulation (Gillard et al. 2018; Lien et al. 2016; Robertson et al. 2017). Atlantic salmon possess more copies of genes encoding LC-PUFA biosynthetic enzymes compared to many other teleosts without recent WGDs (Castro et al. 2012; Monroig et al. 2010; Morais et al. 2009), and this has been hypothesized to have allowed for adaptive evolution of enhanced endogenous lipid synthesis to thrive in the low-dietary LC-PUFA freshwater environment as juveniles (Carmona-Antoñanzas et al. 2014, 2013, 2016). However, evolutionary analyses of genes involved in the LC-PUFA synthesis in Atlantic salmon (Gillard et al. 2018) found that fewer gene duplicates than expected from the genome wide background have been retained after the salmonid WGD in this pathway (Gillard et al. 2018). This does not support a model of adaptive evolution of LC-PUFA synthesis ability through gain in gene copies, yet it does not exclude the possibility that duplicate retention and subsequent evolution of a few key genes have been important. The two Atlantic salmon *elovl5* gene duplicates, for example, are retained and have diverged extensively in regulation (Gillard et al. 2018; Morais et al. 2009). While *elovl5b* is predominantly expressed in the liver, *elovl5a* is mostly expressed in intestinal tissues (Fig. 2). Phylogenetic and functional analyses revealed maintenance of ancestral enzyme activities in both copies of salmon *elovl5* (Carmona-Antoñanzas et al. 2013), but lipid metabolism-regulatory transcription factors have different binding affinities to the promoters of *elovl5a* and *elovl5b* in cellular transfection assays (Carmona-Antoñanzas et al. 2014). Moreover, the two gene copies have different transcriptional responses in vivo upon nutritional changes in Atlantic salmon post-smolts (Morais et al. 2009). It is plausible that this regulatory divergence has allowed for improved adaptive efficiency of elongation steps in the LC-PUFA synthesis.

Another key gene that has been linked to adaptive evolution of LC-PUFA synthesis in other fish is *fads2* (Ishikawa et al. 2019, 2021). There are four *fads2* genes in the Atlantic salmon genome, but these are likely originating from segmental duplications rather than the WGD as they are located on the same chromosome 23 (Lien et al. 2016). Interestingly, sticklebacks that have adapted to low dietary LC-PUFAs in freshwater environments have increased copy numbers of *fads2* genes (Ishikawa et al. 2019, 2021). It is thus possible that more copies of *fads2* genes in Atlantic salmon, and/or divergence of substrate specificities between these copies (∆6-, ∆5-, and ∆8-desaturation activities), have impacted the endogenous biosynthetic capabilities of LC-PUFA in salmon.

## Transcriptional Control of Atlantic Salmon LC-PUFA Biosynthetic Pathways

In this section, we discus transcriptional control of salmon LC-PUFA pathway where we focus on the roles of key transcriptional regulators whose activities are not limited to the regulation of LC-PUFA biosynthesis but extend to other lipid metabolic pathways; the section therefore also covers lipid metabolism in general.

### Characterization of Transcriptional Regulators of Lipid Metabolism in Atlantic Salmon

The major transcription regulators associated with the control of LC-PUFA synthesis in salmon include liver X receptor ( Lxr), Srebp-1, stimulatory protein 1 (Sp1), and nuclear factor Y (NF-Y) (Carmona-Antoñanzas et al. 2014; Zheng et al. 2009). To the best of our knowledge, the Atlantic salmon genome has two copies of *lxr* genes (Cruz-Garcia et al. 2009; Grønvold et al. 2021; Samy et al. 2017). One has been isolated and functionally characterized and it encodes an Lxr-alpha (Lxr-α) protein with 97% and 81% homology to zebrafish and human *LXR*α, respectively (Cruz-Garcia et al. 2009). In post-smolt Atlantic salmon, the *lxrα* gene has highest expression in pyloric caeca and intermediate in liver (Cruz-Garcia et al. 2009), which are both notable sites for active desaturation-elongation of PUFA precursors. Treatment of Atlantic salmon head kidney cells, SHK-1, with GW3965 hydrochloride, a potent and selective Lxr agonist, induced expression of *lxrα* and key LC-PUFA biosynthetic genes, including *∆6fads2-a*, *∆6fads2-b*, and *∆5fads2* (Carmona-Antoñanzas et al. 2014). Furthermore, similar to the mammalian *SREBP-1*c isoform which is a direct target of LXR (Liang et al. 2002; Schultz et al. 2000), GW3965 was shown to induce expression of *srebp-1* in SHK-1 cells (Carmona-Antoñanzas et al. 2014), suggesting a conserved transcriptional regulatory network controlling lipid metabolism between mammals and salmon. It is likely that the salmon Lxrα protein controls LC-PUFA synthesis via activation of *srebp-1*, with Srebp-1 serving as a direct regulator of LC-PUFA biosynthesis. This is supported by findings that Srebp-1 activates the promoters of *elovl5a*, *elovl5b*, and *∆6fads2-a* in Atlantic salmon (Carmona-Antoñanzas et al. 2014). Srebps regulate gene transcription in conjunction with other regulators, such as the NF-Y and Sp1 (Carmona-Antoñanzas et al. 2016). In fact, differences in the magnitude of salmon *elovl5b* promoter activation have been attributed to tandem duplication of both sterol regulatory elements, SREs (recognized and bound by Srebps) and NF-Y binding sites (Carmona-Antoñanzas et al. 2016). It is noteworthy that, while both Srebp-1 and Srebp-2 activate salmon *elovl5a* and *elovl5b* promoters (Carmona-Antoñanzas et al. 2016), only *srebp-1* responds to perturbation in endogenous LC-PUFA synthesis induced by both CRISPR/Cas9-mediated partial knockout of LC-PUFA biosynthetic enzymes and changes in dietary LC-PUFA levels (Datsomor et al. 2019a; Datsomor et al. 2019b; Jin et al. 2020a, b), probably suggesting Srebp-1 rather than Srebp-2 as the main regulator of salmon LC-PUFA biosynthetic pathway. Moreover, results from comparative promoter analysis have shown that Sp1 binding site within salmon *∆6fads2-a* promoter is required for full expression (Zheng et al. 2009), demonstrating the importance of Sp1 in transcriptional control of salmon LC-PUFA biosynthesis. Similarly, Sp1 has been shown to regulate LC-PUFA biosynthesis by upregulating expression of hepatic *∆6/∆5 fads2* and *elovl5* in rabbitfish (*Siganus canaliculatus*) (Li et al. 2019).

PPARs are ligand-dependent transcription factors that belong to the nuclear hormone receptor superfamily. Three isoforms of PPARs have been identified in mammals, namely, PPAR-alpha (PPARα), PPAR-beta or delta (PPARβ or δ), and PPAR-gamma (PPARγ) (Michalik and Wahli 1999). The mammalian PPARs share structural similarities but differ in function, as PPARα is mainly involved in fatty acid oxidation in liver, PPARβ primarily targets adipocyte proliferation, and PPARγ is a master regulator of adipogenesis (Zhou et al. 2015). Four genes encoding PPARβ subtypes have been identified in Atlantic salmon (Leaver et al. 2007) and are grouped into two families (i.e., SsPPARβ1 and SsPPARβ2), each family containing the two isoforms SsPPARβ1A and SsPPARβ1B, and SsPPARβ2A and SsPPARβ2B (Leaver et al. 2007). Only SsPPARβ1A and SsPPARβ2A have been studied functionally. The SsPPARβ1A subtype is predominantly expressed in the liver and it is activated by the mammalian PPARβ-specific ligand GW501516 and monounsaturated fatty acids in contrast to SsPPARβ2A. This suggests central roles for the SsPPARβ1A in fatty acid homeostasis in salmon (Leaver et al. 2007). To the best of our knowledge, PPARs have not demonstrated direct transcriptional control of LC-PUFA synthesis in Atlantic salmon, but PPARα has been shown to increase the activity of *fads2* promoters from rainbow trout and Japanese seabass (Dong et al. 2017). Notably however, salmon PPARα and PPARγ appear to respond to liver phospholipid LC-PUFA levels, where their transcript expression is negatively correlated with hepatic phospholipid 22:6n-3 composition similar to what is observed for Srebp-1 (Jin et al. 2020a, b). Thus, we cannot exclude the involvement of salmon PPARα and PPARγ in the control of LC-PUFA synthesis.

## Nutritional Regulation

### Short-Term Regulation—Impact of Dietary Fatty Acid Manipulation on LC-PUFA Synthesis

Atlantic salmon was traditionally fed fish oil (FO) as the major lipid source in commercial diets; however, since early 1990s, FO has been gradually substituted with plant oil (PO) (Ytrestøyl et al. 2015) partly in a quest to meet the demand for a sustainable salmon aquaculture and also due to shortage of FO as salmon farming steadily expands. As PO is naturally devoid of LC-PUFA, the substitution of dietary FO with PO greatly decreases intake of LC-PUFA, which consequently reduces their tissue content. Salmon fed diet formulated with PO have significant upregulation of liver LC-PUFA biosynthetic genes, including *∆6fads2-a*, *elovl2*, and *∆5fads2* (Morais et al. 2011a, b), with increased fatty acid desaturation and elongation (Bell et al. 2001; Tocher et al. 2003). The PUFA precursors, 18:2n-6 and 18:3n-3, which are abundant in PO have been shown to induce fatty acyl desaturation/elongation in hepatocytes (Sprague et al. 2019; Tocher et al. 2003) albeit low to fully compensate for the loss of dietary LC-PUFA from PO-formulated diets. On the other hand, dietary 20:5n-3 and 22:6n-3, which are enriched in FO, have been shown to negatively correlate with hepatocyte fatty acyl desaturation (Tocher et al. 2003), suggesting a feedback inhibition on the PUFA biosynthetic pathway. The effects of PO and FO are most likely mediated by transcriptional regulators whose targets are genes encoding LC-PUFA biosynthetic enzymes. Dietary fatty acids control transcription regulators via direct binding as ligands or through indirect mechanisms where fatty acids regulate signaling pathways that control gene expression (Jump et al. 2013). In mammals, SREBP-1 has been identified as a key mediator of dietary and ultimately cellular PUFA levels and lipid metabolic gene expression (Ou et al. 2001; Xu et al. 2002, 1999). In the human hepatoma cell, HepG2, media supplemented with n-6 and n-3 LC-PUFA reduce hepatic content of the SREBP-1 protein by 60 and 85%, respectively (Xu et al. 1999). Interestingly, additional experiments revealed that inhibition of SREBP-1 occurs at several levels. Although most inhibition is at the post-transcriptional level (Xu et al. 1999), some may occur at the transcriptional level where LC-PUFA inhibits transcription of the *SREBP-1c* gene by antagonizing ligand-dependent activation of LXR, the upstream regulator of *SREBP-1* (Ou et al. 2001). As mentioned above, Atlantic salmon Srebp-1 is emerging as a mediator of tissue LC-PUFA levels and genes encoding PUFA enzymes. Atlantic salmon fed low dietary LC-PUFA diet showed upregulated transcript levels of *srebp-1* (Datsomor et al. 2019a; Datsomor et al. 2019b; Jin et al. 2020a, b; Morais et al. 2011a, b) and *∆6fads2-a* and *∆5fads2* (Datsomor et al. 2019b; Morais et al. 2012; Morais et al. 2011a, b). On the other hand, SHK-1 cells exposed to cholesterol showed significant upregulation of salmon *srebp-1* while 20:5n-3 and 22:6n-3 treatment reduced *srebp-1*, *∆6fads2-a*, and *∆5fads2* expression (Minghetti et al. 2011). More research is required to elucidate the exact mechanisms by which Srebp-1 mediates dietary LC-PUFA levels and gene expression and determine if this involves other transcription regulatory pathways (e.g., PPARα and PPARγ) other than the Lxr-Srebp-1 pathway which seems to be key to the control of Atlantic salmon LC-PUFA biosynthesis. The importance of the Lxr-Srebp-1 pathway in the regulation of LC-PUFA biosynthesis in rabbitfish has been recently reviewed (Xie et al. 2021). The mechanistic knowledge acquired from various experiments on LC-PUFA biosynthesis and regulation in salmon is illustrated in Fig. 1.

### Life Stage–Associated Changes of LC-PUFA Biosynthetic Pathway

Understanding life stage–associated regulation of LC-PUFA biosynthesis and lipid metabolism in general is key to meeting the nutritional demands of salmon at each stage. An example of such crucial stages is the transitioning from usage of yolk sac to exogenous nutrient resource at first feeding in Atlantic salmon fry. Salmon fry fed a normal commercial diet at first feeding showed significant upregulation of genes of LC-PUFA biosynthetic enzymes in the pyloric caeca (Jin et al. 2019) and in whole fry (Bicskei et al. 2014), probably suggesting a need for LC-PUFA synthesis and utilization at such an early stage. As Atlantic salmon is anadromous, in-depth understanding of changes associated with freshwater and saltwater stages is important to meeting LC-PUFA nutritional requirements. Results from a study which integrated comparative genomics with transcriptomic data from feeding trials across freshwater to saltwater transition showed a striking shift in lipid metabolism after sea water transition, and these include the LC-PUFA biosynthetic pathway (Gillard et al. 2018). The results from this study revealed a concerted shift in metabolic roles of liver and gut after freshwater to saltwater transition, as evident in significant decrease in lipogenic genes including *∆5fads2* and *∆6fads2-a* and the master regulator *srebp-1* in the liver. The gut on the other hand showed increased expression of genes (e.g., apolipoproteins) involved in lipid uptake. Thus, depending on the physiological and metabolic roles, life stage–associated regulation of LC-PUFA pathway may be influenced by the type of organ or tissue, for example, testes of sexually maturing Atlantic salmon males exhibited elevated expression of *∆6fads2-a*, *elovl2*, *elovl5a*, *elovl5b*, and *∆5fads2* compared to immature males (Bogevik et al. 2020), suggesting an important role of LC-PUFA synthesis and utilization during sexual maturation.

## Genetics and LC-PUFA Biosynthesis in Atlantic Salmon: Implication for Increasing LC-PUFA Biosynthetic Capabilities

The genetic background of salmon has been shown to influence both liver fatty acid composition and the expression levels of genes encoding LC-PUFA biosynthetic enzymes (Morais et al. 2011a, b). Furthermore, the content of n-3 LC-PUFA in salmon filet has been identified as a highly heritable trait (Leaver et al. 2011; Horn et al. 2018), with the precursor (18:3n-3) and ultimate product (22:6n-3) of the n-3 LC-PUFA pathway (Fig. 1) showing the highest heritability (Horn et al. 2018). A study conducted by Østbye et al. demonstrated that the genetic background of salmon, specifically the expression levels of *∆6fads2-b*, can significantly influence liver composition of 22:6n-3 and even the sum of PUFAs (Østbye et al. 2021). In this study, progenies of families of Atlantic salmon with average high expression of *∆6fads2-b* showed higher relative levels of liver 22:6n-3 compared to progenies from families with average low *∆6fads2-b* expression (Østbye et al. 2021), underpinning the heritability of LC-PUFA biosynthesis. It is however noteworthy that the correlation between liver and muscle 22:6n-3 seems to be low in salmon (Horn et al. 2019). Thus, more studies encompassing other genes of LC-PUFA biosynthetic enzymes and focusing on filet or muscle LC-PUFA composition are necessary. A recent genome-wide association study in Atlantic salmon identified single nucleotide polymorphisms (SNPs) that have significant association with the ratio of muscle 22:5n-3 and 22:6n-3 (Horn et al. 2020). These genetic variants were located on chromosome 19, close to *elovl2*, which encodes the enzyme that directly elongates 22:5n-3 for synthesis of 22:6n-3 (Fig. 1) (Horn et al. 2020). The identification of these genetic markers associated with LC-PUFA biosynthesis is an essential step towards genetic improvement of salmon LC-PUFA biosynthetic capabilities via selective breeding. A study on land-locked Atlantic salmon population revealed that land-locked salmon parr has higher hepatic expression and activities of desaturase and elongase enzymes compared to farmed salmon (Betancor et al. 2016). Though the higher hepatocyte fatty acyl desaturation and elongation activities did not translate into enhanced flesh n-3 LC-PUFA contents (Betancor et al. 2016), land-locked Atlantic salmon can be a potential genetic resource for improving n-3 LC-PUFA biosynthetic capabilities.

## A Resource for Visualization of Salmon Liver Transcriptomic Data

There is an ever-growing wealth of publicly available transcriptomic data for Atlantic salmon from published studies. It requires time and expertise however to transform this data from a raw to interpretable state. To aid public exploration into this data, we developed an R-script visualization app to aid in explorative and hypothesis-driven analysis of salmon lipid genes. We have processed liver RNAseq data from many liver samples (316) to interactively visualize gene expression data across several studies (Gillard et al. 2018; Jin et al. 2020a, b; Yang Jin et al. 2019). Users may select from curated sets of lipid-related genes along with sets of samples from different experiments and compare gene expression in the auto-generated plot. The app may be used online at https://garethgillard.shinyapps.io/Atlantic_salmon_lipid_expression_viewer or the open code may be accessed from https://gitlab.com/garethgillard/atlantic-salmon-lipid-expression-viewer. See the app documentation for methods of data generation, along with data and method behind Fig. 2.

## Concluding Remarks and Future Perspective

The increasing global demand for fish can potentially be met through sustainable aquaculture, including salmon farming. In the case of the latter, ensuring adequate n-3 LC-PUFA content of farmed salmon is crucial to maintaining its value as a good source of the health-promoting fatty acids, EPA and DHA. In this regard, detailed knowledge about the primary source of n-3 LC-PUFAs in aquafeeds, the endogenous LC-PUFA biosynthetic capabilities, and regulation in salmon is important and needs to be integrated in aquafeed formulation and salmon breeding and farming as a whole. The LC-PUFA biosynthetic capabilities in salmon depend not only on the presence of complementary desaturase and elongase enzymes but also the relatively high functionally redundant and bifunctionality of the multiple enzyme copies partly originating from the salmonid-specific WGD and perhaps also lineage-specific gene duplications. The impact of WGD on the plasticity of salmon LC-PUFA pathway particularly regarding PO-based diets is worth pursuing further to fully understand the evolutionary consequences of WGD on the pathway and also exploit the evolving genome to produce salmon breeds with higher LC-PUFA biosynthetic capability. The transcriptional regulatory networks associated to the LC-PUFA biosynthesis in salmon appear to be similar to what is seen in mammals, in which the Lxr-Srebp-1 pathway is emerging as a central regulator. While several studies acknowledge the key roles of Srebp-1 in salmon, detailed understanding of the interplay between the Lxr-Srebp-1 pathway and dietary as well as endogenously synthesized LC-PUFA is important as this can inform optimal aquafeed formulation.
